# Retinal AI risk stratification and cardiovascular event yield across QRISK3 categories in UK Biobank

**DOI:** 10.1016/j.ajpc.2026.101780

**Published:** 2026-08-03

**Authors:** Sahil Thakur, Jaewon Seo, Zahra Darmani, Dongjin Nam, Husain Khaki, Simon Nusinovici, Tyler Hyungtaek Rim

**Affiliations:** a Mediwhale Inc. Seoul, South Korea; b Al-Farabi Kazakh National University, Almaty, Kazakhstan; c Department of Internal Medicine, Yonsei University College of Medicine, Seoul, South Korea; d Heart Eye Diagnostics Limited, London, United Kingdom; e Duke NUS Medical School, Singapore

**Keywords:** Cardiovascular risk prediction, Retinal imaging, Artificial intelligence, QRISK3, Lipids, UK biobank, Primary prevention

## Introduction

QRISK3 is central to cardiovascular primary prevention in the United Kingdom. NICE recommends estimating 10-year CVD risk in adults aged 25 to 84 without established disease and offering at arranging 20 mg when the estimate is 10% or more [1,2]. The framework works at population scale, but a substantial fraction of future events arises in people below the 10% threshold [[3], [4], [5]]. The practical question for a prevention service is not only who is above a line, but how efficiently a test concentrates true future events among the people it flags. This has prompted interest in complementary biomarkers that capture aspects of vascular biology not represented by traditional risk factors. The retina provides a unique, non-invasive window into the microvasculature, allowing AI algorithms to detect vascular alterations that reflect endothelial dysfunction, microvascular remodeling, and atherosclerotic burden, all of which contribute to cardiovascular disease development [6,7]. Recent advances in artificial intelligence (AI) have enabled the development of retinal imaging biomarkers capable of estimating cardiovascular risk from routine fundus photographs by identifying subtle retinal vascular changes associated with systemic vascular disease [6,[8], [9], [10]]. This study aimed to evaluate whether a retinal AI based cardiovascular risk biomarker provides additional risk enrichment beyond QRISK3 in the UK Biobank cohort, with a particular focus on identifying individuals at higher observed cardiovascular risk within established risk strata.

## Methods

This was a retrospective analysis of UK Biobank (approved application 68,428), restricted to participants with a gradable fundus photograph (image quality score below 0.2), no prevalent CVD at baseline, no lipid-lowering therapy at baseline and the inputs required to compute QRISK3 (n = 49,611). Baseline was the retinal imaging date and follow-up extended up to 10 years. (Fig. 1A) The outcome was the first fatal or non-fatal CVD event within 10 years, defined by ICD-10 codes for ischaemic heart disease (I20 to I25), heart failure (I50), cerebrovascular disease (I60 to I69), transient ischaemic attack (G45) and hypertensive cardiovascular disease (I11.0, I13.0, I13.2, I12.1), from linked hospital and mortality records. QRISK3 was computed from UK Biobank baseline data by complete-case analysis, using measured demographic, blood-pressure, body-mass-index and lipid values and self-reported smoking, diabetes, family history and comorbidities.

**Fig. 1:**
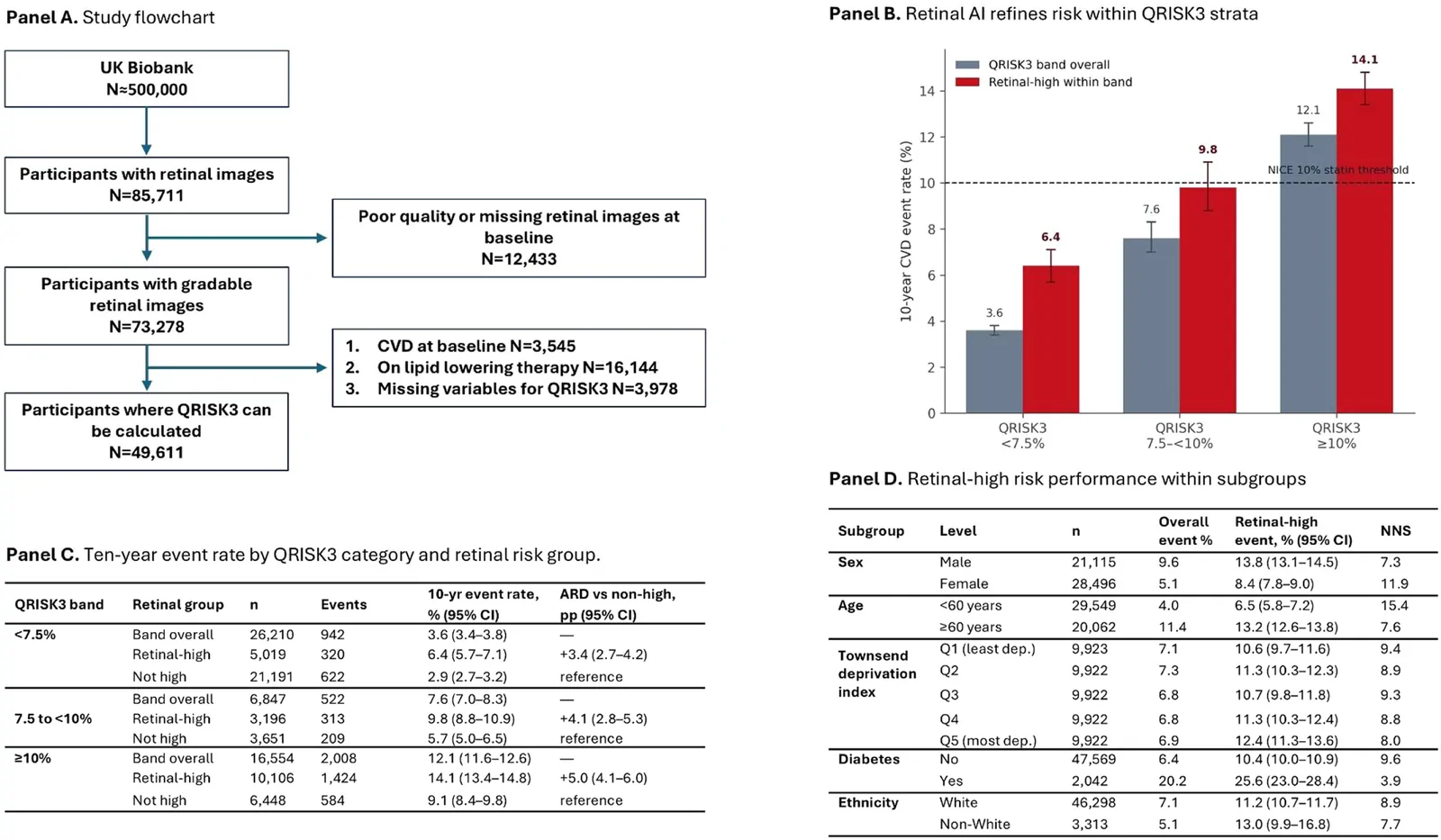
**Retinal AI based cardiovascular risk enrichment across QRISK3 risk categories in UK Biobank. (A)** Flow diagram showing derivation of the UK Biobank analytic cohort. **(B)** Ten year cardiovascular event rates across QRISK3 categories, comparing the overall cohort with participants classified as retinal high risk. **(C)** Ten year cardiovascular event rates by QRISK3 category and retinal risk group, with absolute risk differences and 95% confidence intervals. **(D)** Retinal high risk event rates and number needed to screen across demographic and clinical subgroups. **Abbreviations:** AI, artificial intelligence; ARD, absolute risk difference; CI, confidence interval; CVD, cardiovascular disease; NICE, National Institute for Health and Care Excellence; NNS, number needed to screen; pp, percentage points; QRISK3, United Kingdom QRISK3 cardiovascular disease risk prediction algorithm.

The retinal AI biomarker (Dr. Noon CVD version 1.0.0, previously known as Reti-CVD; Mediwhale Inc., Seoul, Republic of Korea) is a deep convolutional neural network trained on fundus photographs paired with CT-measured coronary artery calcium (CAC) and outputs a continuous risk score. The score was categorized into low, moderate and high risk using the prespecified strata in the algorithm’s pivotal trial [7]. High risk corresponds to a score above 0.4074 and moderate risk to 0.3093–0.4074 on the native 0 to 1 scale (equivalently, cut-offs of 31 and 41 on a 0 to 100 scale). These thresholds were fixed before the present analysis and were not re-optimized; UK Biobank was not used in model development, threshold selection or tuning [6,7]. We compared three stratification approaches: untreated LDL-C thresholds (2.6, 3.0, 4.9 mmol/L), QRISK3 categories (<7.5%, 7.5 to <10%, and 10% or more, the last matching the NICE statin threshold) and the retinal high-risk group, each alone and in combination using a restrictive rule requiring both criteria positive. For each rule, treated as a binary classifier of 10-year events, we computed the event rate, sensitivity, specificity, PPV and NNS, each with 95% Wilson confidence intervals (CIs); absolute risk differences used the Wald method. We also report discrimination (Harrell C-index) and a sensitivity analysis restricting the endpoint to ASCVD-type events. Model architecture, training and prior external validation are described in the cited reports.

## Results

The analytic cohort comprised 49,611 untreated participants (mean age 56.0 [SD 8.3] years, 57.4% female, 93.3% White; mean retinal score 37.0 [SD 9.1]; mean QRISK3 8.5% [SD 6.3], mean LDL-C 3.70 [SD 0.81] mmol/L). The retinal model classified 25.3% as low, 37.8% as moderate and 36.9% as high risk, and QRISK3 placed 52.8% below 7.5%, 13.8% in the borderline band and 33.4% at 10% or more. Over 10 years there were 3472 incident CVD events (7.0%). Event rates rose across retinal categories, from 2.7% (95% CI 2.4–3.0) in the low group to 5.7% (5.4–6.1) moderate to 11.2% (10.8–11.7) high; the high group (2057 events among 18,321) was similar to QRISK3 of 10% or more (12.1%, 11.6–12.6; 2008 among 16,554), and discrimination was comparable for the two (C-index 0.64 and 0.65) and weak for untreated lipid thresholds (C-index 0.51–0.52). As a standalone classifier the retinal high-risk group reached a PPV of 11.2% (10.8–11.7), a sensitivity of 59.2% (57.6–60.9), a specificity of 64.7% (64.3–65.2) and an NNS of 8.9 (8.6–9.3); QRISK3 of 10% or more performed similarly (PPV 12.1% [11.6–12.6], sensitivity 57.8% [56.2–59.5], specificity 68.5% [68.0–68.9], NNS 8.2 [7.9–8.6]). Untreated lipid thresholds separated events weakly (PPV 7.1% to 9.2%; NNS 10.8–14.0). Within each QRISK3 band the retinal high-risk subgroup had a higher observed event rate than the band overall (Fig. 1B) and the non-high subgroup in the same band, with an absolute risk difference of 3.4 percentage points (95% CI 2.7–4.2) below 7.5%, 4.1 (2.8–5.3) in the borderline band and 5.0 (4.1–6.0) at 10% or more; denominators, events and event rates are in the Fig. 1 data table (Fig. 1C).

Requiring both retinal high risk and QRISK3 of 10% or more produced the highest yield of any strategy (PPV 14.1%, 13.4–14.8; NNS 7.1, 6.8–7.4) but flagged fewer people, so sensitivity fell to 41.0% (39.4–42.7). This is a trade-off, not an unambiguous gain: yield rose while event capture fell. Reclassification relative to the statin threshold was limited. Of the 18,321 retinal high-risk participants, 8215 were below QRISK3 10%; their observed 10-year event rate was 7.7% (7.1–8.3), which remained below the NICE 10% threshold, while 6448 participants who were QRISK3 of 10% or more, but not retinal high risk had an observed rate of 9.1% (8.4–9.8). In every subgroup (sex, age, Townsend deprivation, diabetes and ethnicity) the retinal high-risk event rate exceeded the subgroup overall rate (Fig. 1D); the highest absolute rates were in participants with diabetes (retinal high-risk 25.6% versus 20.2% overall). In a sensitivity analysis restricting the endpoint to ASCVD-type events (myocardial infarction and other ischaemic heart disease, ischaemic stroke, transient ischaemic attack and cardiovascular death, excluding heart failure and hypertensive cardiovascular disease), 3212 of the 3472 composite events qualified (6.5% of the cohort) and estimates were directionally consistent but modestly attenuated: the retinal high-risk group had a 10-year ASCVD event rate of 10.3% (1886/18,321) versus 11.1% for QRISK3 of 10% or more, within-band enrichment persisted (retinal high-risk 5.7%, 8.7% and 12.9% across the <7.5%, 7.5–<10% and ≥10% bands), and the combined retinal-plus-QRISK3 rule again raised PPV from the 6.5% cohort rate to 12.9% (12.3–13.5) with sensitivity 41.8% (40.1–43.6).

## Discussion

In this untreated UK Biobank cohort, a CAC-trained retinal AI biomarker identified subgroups with higher observed 10-year CVD event rates within every QRISK3 category, and the retinal high-risk group reached a PPV and discrimination similar to QRISK3 of 10% or more (C-index 0.64 versus 0.65). The enrichment signal is consistent across bands and precisely estimated. Its incremental clinical value, however, is modest, and there are observations that merit consideration. First, the borderline group remained below the 10% treatment threshold even after retinal enrichment (7.6% to 9.8%), and the 8215 people who were retinal high risk but below QRISK3 10% had an event rate of 7.7%, so on current NICE rules this degree of enrichment would not by itself move most sub-threshold patients into statin eligibility. Second, higher PPV from the combined rule was achieved by narrowing the flagged population: the retinal-plus-QRISK3 rule raised PPV to 14.1% but captured only 41.0% of events, so higher yield came at the cost of event capture. These are association analyses in a single cohort, not a test of whether retinal-guided treatment changes outcomes; whether the enrichment signal translates into clinical benefit remains unproven.

Limitations include a single, predominantly European-ancestry cohort subject to healthy-volunteer selection, so absolute event rates and yields may not transfer to the general population. QRISK3 was computed from UK Biobank baseline data, which differs from routine primary-care records in how some variables are recorded and has a healthy volunteer bias [11] Diabetic retinopathy screening is an opportunistic setting where fundus images are already captured and could provide an estimate of CVD risk; the present cohort excluded prevalent CVD and baseline lipid-lowering therapy and was not a diabetic screening population, which we note as a limitation. Formal calibration to absolute risk, decision-curve analysis and net reclassification analyses were not undertaken here and are needed, alongside external validation and prospective evaluation, before clinical use.

## Conclusion

In a selected UK Biobank cohort with retinal imaging, an AI biomarker identified subgroups with higher 10-year CVD event rates within QRISK3 categories and achieved PPV similar to QRISK3 of 10% or more. These findings support further evaluation of retinal AI as a risk-enrichment tool, but external validation, calibration, decision-curve analysis and prospective implementation studies are needed before clinical adoption.

## Disclosure

ST is an employee of Mediwhale Inc.

JS is an employee of Mediwhale Inc.

DN is an employee of Mediwhale Inc.

HK is an employee of Heart Eye Diagnostics Limited.

SN has served as consultant to Mediwhale Inc. and has received consultation fees from the company.THR is an employee of Mediwhale Inc., owns stock in the company, and holds the following patents that may be affected by this study: 10-2018-0166720 (KR), 10-2018-0166721 (KR), 10-2018-0166722 (KR), 62/694,901 (US), 62/715,729 (US), and 62/776,345 (US).

All other authors declare no competing interests.

## Funding

This work was supported by the Technological Innovation R&D Program (SCALEUP TIPS), funded by the Ministry of SMEs and Startups (MSS, Korea) (Grant No. RS-2025-25465723).

## CRediT authorship contribution statement

**Sahil Thakur:** Writing – review & editing, Writing – original draft, Visualization, Methodology, Investigation, Formal analysis, Data curation, Conceptualization. **Jaewon Seo:** Visualization, Validation, Methodology, Formal analysis. **Zahra Darmani:** Writing – review & editing, Writing – original draft. **Dongjin Nam:** Writing – review & editing. **Husain Khaki:** Writing – review & editing. **Simon Nusinovici:** Visualization, Validation, Methodology, Formal analysis. **Tyler Hyungtaek Rim:** Writing – review & editing, Validation, Supervision, Software, Project administration, Methodology, Funding acquisition, Conceptualization.

## Declaration of competing interest

The authors declare the following financial interests/personal relationships which may be considered as potential competing interests: Sahil Thakur reports financial support was provided by Mediwhale Inc. Jaewon Seo reports financial support was provided by Mediwhale Inc. Dongjin Nam reports financial support was provided by Mediwhale Inc. Husain Khaki reports financial support was provided by Heart Eye Diagnostics Limited. Tyler Hyungtaek Rim reports financial support was provided by Mediwhale Inc. Simon Nusinovici reports a relationship with Mediwhale Inc. that includes: consulting or advisory and employment. Tyler Hyungtaek Rim reports a relationship with Mediwhale Inc. that includes: equity or stocks. Tyler Hyungtaek Rim has patent #10-2018-0166,720 (KR) issued to Mediwhale Inc. Tyler Hyungtaek Rim has patent #10-2018-0166,721 (KR) issued to Mediwhale Inc. Tyler Hyungtaek Rim has patent #10-2018-0166,722 (KR) issued to Mediwhale Inc. Tyler Hyungtaek Rim has patent #62/694,901 (US) issued to Mediwhale Inc. Tyler Hyungtaek Rim has patent #62/715,729 (US) issued to Mediwhale Inc. Tyler Hyungtaek Rim has patent #62/776,345 (US) issued to Mediwhale Inc. If there are other authors, they declare that they have no known competing financial interests or personal relationships that could have appeared to influence the work reported in this paper.
